# Quantitative analysis of acetyl-CoA production in hypoxic cancer cells reveals substantial contribution from acetate

**DOI:** 10.1186/2049-3002-2-23

**Published:** 2014-12-11

**Authors:** Jurre J Kamphorst, Michelle K Chung, Jing Fan, Joshua D Rabinowitz

**Affiliations:** Lewis-Sigler Institute for Integrative Genomics, Carl Icahn Laboratory, Princeton University, Princeton, NJ 08544 USA; Cancer Research UK Beatson Institute & Institute of Cancer Sciences, University of Glasgow, Garscube Estate, Switchback Road, Glasgow, G61 1BD UK

**Keywords:** Acetate, Acetyl-CoA, Cancer metabolism, Fatty acids, Hypoxia, Lipogenesis, Mass spectrometry, Palmitate, ^13^C-tracing

## Abstract

**Background:**

Cell growth requires fatty acids for membrane synthesis. Fatty acids are assembled from 2-carbon units in the form of acetyl-CoA (AcCoA). In nutrient and oxygen replete conditions, acetyl-CoA is predominantly derived from glucose. In hypoxia, however, flux from glucose to acetyl-CoA decreases, and the fractional contribution of glutamine to acetyl-CoA increases. The significance of other acetyl-CoA sources, however, has not been rigorously evaluated. Here we investigate quantitatively, using ^13^C-tracers and mass spectrometry, the sources of acetyl-CoA in hypoxia.

**Results:**

In normoxic conditions, cultured cells produced more than 90% of acetyl-CoA from glucose and glutamine-derived carbon. In hypoxic cells, this contribution dropped, ranging across cell lines from 50% to 80%. Thus, under hypoxia, one or more additional substrates significantly contribute to acetyl-CoA production. ^13^C-tracer experiments revealed that neither amino acids nor fatty acids are the primary source of this acetyl-CoA. Instead, the main additional source is acetate. A large contribution from acetate occurs despite it being present in the medium at a low concentration (50–500 μM).

**Conclusions:**

Acetate is an important source of acetyl-CoA in hypoxia. Inhibition of acetate metabolism may impair tumor growth.

**Electronic supplementary material:**

The online version of this article (doi:10.1186/2049-3002-2-23) contains supplementary material, which is available to authorized users.

## Background

Cancer cells have genetic mutations that drive proliferation. Such proliferation creates a continuous demand for structural components to produce daughter cells [1–3]. This includes demand for fatty acids for lipid membranes. Cancer cells can obtain fatty acids both through uptake from extracellular sources and through *de novo* synthesis, with the latter as a major route by which non-essential fatty acids are acquired in many cancer types [4, 5].

The first fatty acid to be produced by *de novo* fatty acid synthesis is palmitate. The enzyme fatty acid synthase (FAS) makes palmitate by catalyzing the ligation and reduction of 8-acetyl (2-carbon) units donated by cytosolic acetyl-CoA. This 16-carbon fatty acid palmitate is then incorporated into structural lipids or subjected to additional elongation (again using acetyl-CoA) and desaturation reactions to produce the diversity of fatty acids required by the cell.

Acetyl-CoA sits at the interface between central carbon and fatty acid metabolism. In well-oxygenated conditions with abundant nutrients, its 2-carbon acetyl unit is largely produced from glucose. First, pyruvate dehydrogenase produces acetyl-CoA from glucose-derived pyruvate in the mitochondrion, followed by ligation of the acetyl group to oxaloacetate to produce citrate. Citrate is then transported into the cytosol and cytosolic acetyl-CoA produced by ATP citrate lyase.

In hypoxia, flux from glucose to acetyl-CoA is impaired. Low oxygen leads to the stabilization of the HIF1 complex, blocking pyruvate dehydrogenase (PDH) activity via activation of HIF1-responsive pyruvate dehydrogenase kinase 1 (PDK1) [6, 7]. As a result, the glucose-derived carbon is shunted towards lactate rather than being used for generating acetyl-CoA, affecting carbon availability for fatty acid synthesis.

To understand how proliferating cells rearrange metabolism to maintain fatty acid synthesis under hypoxia, multiple studies focused on the role of glutamine as an alternative carbon donor [8–10]. The observation that citrate M^+5^ labeling from U-^13^C-glutamine increased in hypoxia led to the hypothesis that reductive carboxylation of glutamine-derived α-ketoglutarate enables hypoxic cells to maintain citrate and acetyl-CoA production. As was noted later, though, dropping citrate levels in hypoxic cells make the α-ketoglutarate to citrate conversion more reversible and an alternative explanation of the extensive citrate and fatty acid labeling from glutamine in hypoxia is isotope exchange without a net reductive flux [11]. Instead, we and others found that hypoxic cells can at least in part bypass the need for acetyl-CoA for fatty acid synthesis by scavenging serum fatty acids [12, 13].

In addition to increased serum fatty acid scavenging, we observed a large fraction of fatty acid carbon (20%–50% depending on the cell line) in hypoxic cells not coming from either glucose or glutamine. Here, we used ^13^C-tracers and mass spectrometry to quantify the contribution from various carbon sources to acetyl-CoA and hence identify this unknown source. We found only a minor contribution of non-glutamine amino acids and of fatty acids to acetyl-CoA in hypoxia. Instead, acetate is the major previously unaccounted for carbon donor. Thus, acetate assimilation is a route by which hypoxic cells can maintain lipogenesis and thus proliferation.

## Methods

### Cell culture and isotope tracing

All cell lines were from ATCC, were routinely passaged in Dulbecco’s Modified Eagle Medium (DMEM; Mediatech) with 25 mM glucose and 4 mM glutamine, were supplemented with 10% fetal bovine serum (HyClone), 25 IU/ml penicillin, and 25 μg/ml streptomycin (MP Biomedicals), and were split at 80% confluence. Metabolic experiments were performed in 6-cm culture dishes with 3 ml of DMEM containing 10% dialyzed serum (DFBS; HyClone). For isotope labeling experiments, glucose and/or amino acids were replaced as indicated with their U-^13^C-labeled forms (Cambridge Isotope Labs). U-^13^C-acetate was spiked into the medium to achieve indicated concentrations. U-^13^C-palmitate was complexed to fatty-acid-free BSA (Roche) in 6:1 molar ratio in a 150 mM NaCl solution by stirring overnight at 37°C and provided to cells to the indicated concentration. For all ^13^C-tracing experiments, cells were maintained in the labeled medium for 48 h unless otherwise indicated. Medium acetate was quantified by colorimetric assay (BioVision). Hypoxia experiments were performed in a hypoxic glove box (1% O_2_, 5% CO_2_, and 94%–94.5% N_2_, at 37°C) (Coy Laboratory Products). Cells and media were equilibrated in low oxygen overnight before experiment initiation.

### Sample preparation and analysis

For analysis of saponified fatty acids, the media were aspirated, cells rinsed twice with 2 ml room-temperature PBS, 1 ml 50:50 MeOH/H_2_O solution with 0.1 M HCl at −20°C added, and the resulting liquid and cell debris scraped into a microfuge tube. Chloroform (0.5 ml) was added, the mixture was vortexed for 1 min and then centrifuged at 16,000 × *g* for 5 min, and the chloroform layer was transferred to a glass vial. The extract was dried under N_2_, reconstituted into 90:10 MeOH/H_2_O containing 0.3 M KOH, incubated at 80°C for 1 h to saponify the fatty acids, acidified with 0.1 ml of formic acid, extracted twice with 1 ml hexane, dried under N_2_, and reconstituted into 1:1:0.3 MeOH:chloroform:H_2_O (1 ml solvent per 1 × 10^6^ cells) for liquid chromatography–mass spectrometry (LC-MS) analysis. Separation was by reversed-phase ion-pairing chromatography on a C8 column coupled to negative-ion mode full-scan LC-MS at 1 Hz scan time and 100,000 resolving power (stand-alone orbitrap, Thermo Fisher Scientific) [14]. Subsequent peak integration and computation were done with MAVEN and MATLAB (MathWorks), respectively [15].

## Results

### Cytosolic acetyl-CoA labeling can be inferred from fatty acid labeling patterns

**Figure 1:**
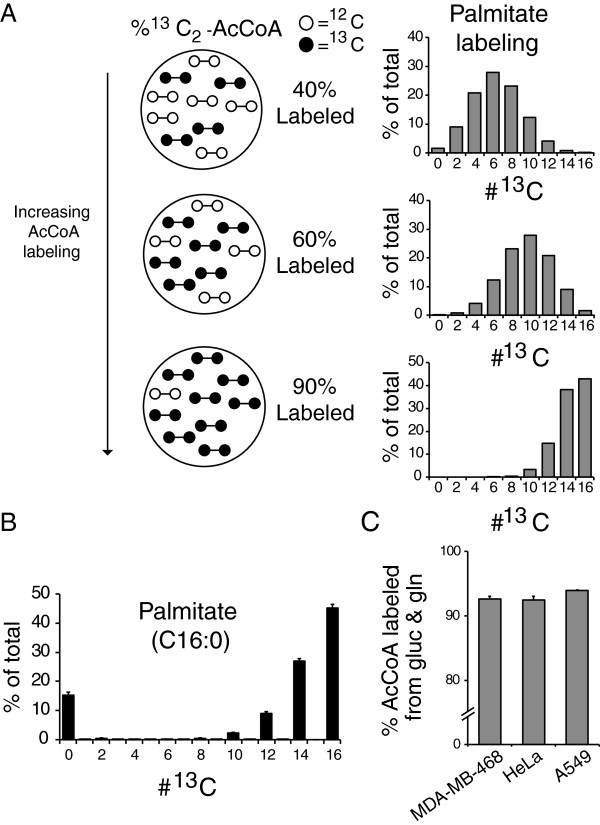
**Percentage** ^**13**^ **C-labeling of cytosolic acetyl-CoA can be quantified from palmitate labeling. (A)** Increasing ^13^C_2_-acetyl-CoA labeling shifts palmitate labeling pattern to the right. ^13^C_2_-acetyl-CoA labeling can be quantified by determining a best fit between observed palmitate labeling and computed binomial distributions (shown on right-hand side) from varying fractions of acetyl-CoA (AcCoA) labeling. **(B)** Steady-state palmitate labeling from U-^13^C-glucose and U-^13^C-glutamine in MDA-MB-468 cells. **(C)** Percentage acetyl-CoA production from glucose and glutamine. For (B) and (C), data are means ± SD of *n* = 3.



Proliferating cancer cells make a significant fraction of non-essential fatty acids *de novo* [12]. This is done by successive ligation and reduction steps of 2-carbon acetyl units donated by cytosolic acetyl-CoA (AcCoA). AcCoA can be produced from various substrates including glucose, amino acids, and fatty acids. Quantitative assessment of AcCoA production can be performed by feeding ^13^C-labeled substrates followed by a direct analysis of AcCoA labeling by mass spectrometry [16]. However, AcCoA labeling can also be inferred from fatty acids [8, 9]. This provides several advantages: fatty acids are more stable and abundant than AcCoA and their labeling specifically reflects labeling of cytosolic AcCoA. Labeling of the 16-carbon-saturated fatty acid palmitate can be reliably measured by LC-MS [14], with increased AcCoA labeling causing a shift to the right in the labeling distribution (Figure 1A). The frequency of each labeled form (after correction for natural ^13^C abundance) reflects a binomial distribution from which the fractional acetyl labeling of cytosolic AcCoA (*p*) can be quantified by minimizing the sum of squared residuals between the calculated and experimentally observed palmitate labeling:

We applied this approach to MDA-MB-468 cells grown in medium containing U-^13^C-glucose and U-^13^C-glutamine. The resulting steady-state palmitate labeling patterns showed multiple heavily ^13^C-labeled forms as well as a remaining unlabeled M^0^ peak (Figure 1B). The M^0^-labeled form results from scavenging of unlabeled serum fatty acids and can be disregarded for the purpose of determining AcCoA labeling. From the remaining labeling distribution, we calculated 87% AcCoA labeling from glucose and 6% from glutamine, with 93% collectively accounted for by these two major carbon sources (Additional file 1: Figure S1). Similar results were also obtained for HeLa and A549 cells (Figure 1C).

### A substantial fraction of cytosolic AcCoA does not come from glucose or glutamine in hypoxic cells

**Figure 2:**
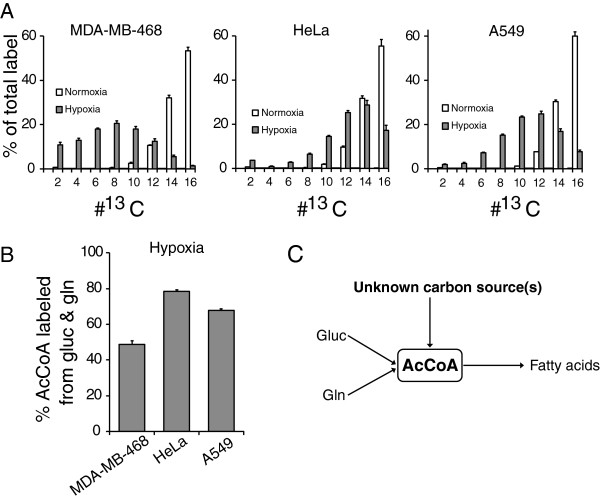
**Acetyl-CoA labeling from** ^**13**^ **C-glucose and** ^**13**^ **C-glutamine decreases in hypoxia. (A)** Steady-state palmitate labeling from U-^13^C-glucose and U-^13^C-glutamine in normoxic and hypoxic (1% O_2_) conditions. **(B)** Percentage acetyl-CoA production from glucose and glutamine in hypoxia. **(C)** One or more additional carbon donors contribute substantially to acetyl-CoA production in hypoxia. Abbreviations: Gluc, glucose; Gln, glutamine. Data are means ± SD of *n* = 3.

Hypoxia is a common occurrence in tumors [17–19] and affects central carbon metabolism [6, 7]. To investigate how hypoxia impacts cytosolic acetyl-CoA production, we cultured cells in 1% O_2_ in the presence of U-^13^C-glucose, U-^13^C-glutamine, and with both substrates labeled, followed by LC-MS analysis of palmitate labeling. Analogous to earlier observations, we noticed decreased labeling of AcCoA from glucose and increased labeling from glutamine in hypoxia (Additional file 1: Figure S1) [8–10]. Strikingly, however, in experiments where both glucose and glutamine were fully ^13^C-labeled, there was a shift in palmitate labeling distribution towards the left (i.e., towards more unlabeled AcCoA) for hypoxic MDA-MB-468 cells compared to normoxic cells (Figure 2A). We made comparable observations (albeit somewhat less pronounced) in HeLa and A549 cells. Quantifying AcCoA labeling from palmitate demonstrated that between 49% of AcCoA in MDA-MB-468 cells and 78% in HeLa cells labeled from glucose and glutamine (Figure 2B), whereas in normoxic conditions the labeling exceeded 90% for all cells. Therefore, hypoxia significantly affected AcCoA production, with ~20%–50% of the AcCoA pool being derived from one or more carbon donors other than glucose and glutamine (Figure 2C).

### Amino acids and fatty acids are minor contributors to AcCoA

**Figure 3:**
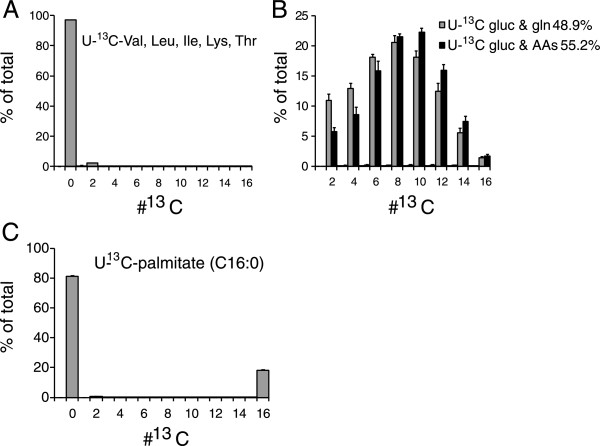
**Amino acids (other than glutamine) and fatty acids are not major sources of cytosolic acetyl-CoA in hypoxia. (A)** Palmitate labeling in hypoxic (1% O_2_) MDA-MB-468 cells, grown for 48 h in medium where branched chain amino acids plus lysine and threonine were substituted with their respective U-^13^C-labeled forms. **(B)** Same conditions, except that glucose and glutamine only or glucose and all amino acids, were substituted with the U-^13^C-labeled forms. **(C)** Palmitate labeling in hypoxic (1% O_2_) MDA-MB-468 cells, grown in medium supplemented with 20 μM U-^13^C-palmitate for 48 h. Data are means ± SD of *n* = 3.

Catabolism of other medium components beyond glucose and glutamine must contribute substantially to the AcCoA pool in hypoxia. We identified amino acids as likely candidates and first tested amino acids whose breakdown directly leads to AcCoA, i.e., the branched chain amino acids, lysine, and threonine [20]. For this, we cultured MDA-MB-468 cells at 1% O_2_ in medium analogous to DMEM but with the U-^13^C-labeled forms of the indicated amino acids. Virtually no ^13^C-labeling was observed in palmitate (Figure 3A), indicating that these amino acids were not major contributors to AcCoA production. To rule out potential contributions from other amino acids, we cultured the cells in DMEM with U-^13^C-labeled forms of glucose and all amino acids. A modest increase was observed in AcCoA labeling compared to DMEM with U-^13^C-glucose and U-^13^C-glutamine (Figure 3B), but this accounted for only a minor fraction of the alternative AcCoA source.

As DMEM components appeared not to be responsible for AcCoA production and the cells were cultured in the presence of 10% serum, we considered lipid/fatty acid oxidation as a possible AcCoA source [21]. We incubated cells with 20 μM U-^13^C-palmitate, which led to approximately 20% labeling of the total cellular palmitate pool (Figure 3C). Oxidation of the labeled palmitate and subsequent AcCoA production should lead to synthesis of partially labeled forms of palmitate, particularly the M^+2^ and M^+4^ forms (Additional file 1: Figure S2). For example, given that 20% of the cellular palmitate pool is fully labeled, even if just 13% of the contribution from the unidentified source were derived from palmitate oxidation, a 10% M^+2^ form should be present. Only a much smaller M^+2^ peak was observed. Although selected lipids other than palmitate might still contribute to the AcCoA pool, the simplest interpretation is that fatty acid oxidation is not the primary unidentified AcCoA source.

### Acetate is the main additional AcCoA carbon source in hypoxia

**Figure 4:**
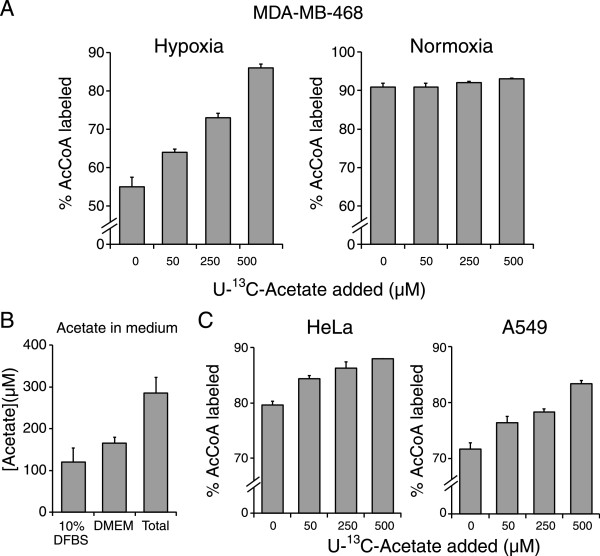
**The main additional AcCoA source in hypoxia is acetate. (A)** Percentage ^13^C_2_-acetyl-CoA labeling quantified from palmitate labeling in hypoxic (1% O_2_) and normoxic MDA-MB-468 cells grown in medium with U-^13^C-glucose and U-^13^C-glutamine and additionally supplemented with indicated concentrations of U-^13^C-acetate. **(B)** Acetate concentrations in fresh 10% DFBS, DMEM, and DMEM with 10% DFBS. **(C)** Percentage ^13^C_2_-acetyl-CoA labeling for hypoxic (1% O_2_) HeLa and A549 cells. For (A) and (C), data are means ± SD of *n* ≥ 2. For (B), data are means ± SEM of *n* = 3.

We next investigated if hypoxic cells could activate acetate to AcCoA. Although we used dialyzed serum in our experiments and acetate is not a component of DMEM, we contemplated the possibility that trace levels could still be present or that acetate is produced as a catabolic intermediate from other sources (for example from protein de-acetylation). We cultured MDA-MB-468 cells in 1% O_2_ in DMEM containing U-^13^C-glucose and U-^13^C-glutamine and added increasing amounts of U-^13^C-acetate (Figure 4A). AcCoA labeling rose considerably with increasing U-^13^C-acetate concentrations, from approximately 50% to 86% with 500 μM U-^13^C-acetate. No significant increase in labeling of AcCoA was observed in normoxic cells following incubation with U-^13^C-acetate. Thus, acetate selectively contributes to AcCoA in hypoxia.

To determine if trace acetate levels in the culture medium could account for the missing carbon source or that catabolic reactions producing acetate were more likely, we measured acetate levels in fresh DMEM without serum, in dialyzed serum, and in fresh DMEM with 10% dialyzed serum (Figure 4B). Strikingly, both DMEM and serum (although dialyzed) contained considerable acetate, with a total of approximately 285 μM in the complete culture medium (DMEM with 10% dialyzed serum). This is within the range of reported plasma acetate concentrations in human subjects (50–650 μM) [22–25]. Adding 250 μM U-^13^C-acetate to this medium caused an 18% increase in AcCoA labeling to a total of 73% (Figure 4A). Considering that the labeled and unlabeled fractions in this particular condition were roughly equal, this would translate to 91% AcCoA labeling if the entire acetate pool were to be labeled, similar to AcCoA labeling from glucose and glutamine in normoxic cells (Figure 1C). Therefore, the acetate present in fresh culturing medium appears to be the main hypoxia-induced contributor to AcCoA. Similar to MDA-MB-468 cells, U-^13^C-acetate also labels AcCoA in hypoxic HeLa and A549 cells (Figure 4C).

## Discussion

Tumors require a constant supply of fatty acids to sustain cellular replication. It is thought that most cancers derive a considerable fraction of the non-essential fatty acids through *de novo* synthesis. This requires AcCoA with its 2-carbon acetyl group acting as the carbon donor. In nutrient replete and well-oxygenated conditions, AcCoA is predominantly made from glucose. However, tumor cells often experience hypoxia, causing limited entry of glucose-carbon into the TCA cycle. This in turn affects AcCoA production, and it has been proposed that hypoxic cells can compensate by increasing AcCoA production from glutamine-derived carbon in a pathway involving reductive carboxylation of α-ketoglutarate [8–10].

Here we studied carbon incorporation from ^13^C-labeled substrates into palmitate [14], using partial palmitate labeling to derive cytosolic AcCoA labeling. Consistent with earlier findings [8–10], we observed increasing AcCoA labeling from glutamine in hypoxia. A substantial fraction of AcCoA, however, did not label from glucose or glutamine. Through subsequent ^13^C-tracing studies, we found that this fraction labels from acetate.

It is important to differentiate between a substrate’s contribution to AcCoA labeling and net synthesis. A high labeling fraction can potentially be achieved by a substrate that exchanges (or forms an intermediate that exchanges) 2-carbon units back and forth with AcCoA (or with its parent molecule citrate), without necessarily making a net contribution to the AcCoA pool. With Tomer Shlomi, we have argued that an exchange between α-ketoglutarate and citrate, rather than net 2-carbon unit donation, explains most AcCoA labeling from glutamine [11]. Formation of AcCoA from acetate is mediated by acetyl-CoA synthetases (ACSS1 is mitochondrial and ACSS2 is cytosolic), which consume ATP to drive the net reaction; thus, a reversible exchange between acetate and AcCoA is unlikely to explain the observed AcCoA labeling from acetate in hypoxia. Nevertheless, it is possible that a futile cycling between acetate and AcCoA inflates the apparent acetate contribution. Other factors, however, may lead to an underestimation of this contribution. AcCoA labeling from U-^13^C-glucose may occur via free labeled acetate as an intermediate. AcCoA labeling from U-^13^C-glutamine may involve a reversible exchange between α-ketoglutarate and citrate that dilutes labeling from acetate. Thus, it is likely that the contribution of acetate to net AcCoA synthesis actually exceeds the extent of labeling observed here.

Irrespective of the precise net contribution of acetate in hypoxia, a remarkable aspect is that a significant contribution occurs based only on contaminating acetate (~300 μM) in the culturing medium. This is considerably less than glucose (25 mM) or glutamine (4 mM). Acetate concentrations in the plasma of human subjects have been reported in the range of 50 to 650 μM [22–25], and therefore, significant acetate conversion to AcCoA may occur in human tumors. This is supported by clinical observations that ^11^C-acetate PET can be used to image tumors, in particular those where conventional FDG-PET typically fails [26]. Our results indicate that ^11^C-acetate PET could be particularly important in notoriously hypoxic tumors, such as pancreatic cancer. Preliminary results provide evidence in this direction [27].

Finally, as our measurements of fatty acid labeling reflect specifically cytosolic AcCoA, it is likely that the cytosolic acetyl-CoA synthetase ACSS2 plays an important role in the observed acetate assimilation. Accordingly, inhibition of ACSS2 merits investigation as a potential therapeutic approach.

## Conclusions

In hypoxic cultured cancer cells, one-quarter to one-half of cytosolic acetyl-CoA is not derived from glucose, glutamine, or other amino acids. A major additional acetyl-CoA source is acetate. Low concentrations of acetate (e.g., 50–650 μM) are found in the human plasma and also occur as contaminants in typical tissue culture media. These amounts are avidly incorporated into cellular acetyl-CoA selectively in hypoxia. Thus, ^11^C-acetate PET imaging may be useful for probing hypoxic tumors or tumor regions. Moreover, inhibiting acetate assimilation by targeting acetyl-CoA synthetases (e.g., ACSS2) may impair tumor growth.

## Electronic supplementary material

Additional file 1: Figure S1: Percent labeling of acetyl groups from U-^13^C-glucose (Gluc) and U-^13^C-glutamine (Gln) in normoxia and hypoxia (1% O_2_). Acetyl labeling from *N*-acetyl-glutamate and glutamate at a steady state; analysis of fatty acid labeling gives equivalent results. All data are mean ± SD of *N* = 3. **Figure S2.** Calculation of expected palmitate labeling, based on the observation that 20% of palmitate pool is U-^13^C-labeled, for palmitate oxidation contributing ‘X’ percent to the fraction of AcCoA not derived from glucose or glutamine. Even if X is 13%, significant M + 2 labeling would be expected, which was not observed experimentally (see Figure 3). (PDF 136 KB)
