# Supplementary material for: Quantitative analysis of acetyl-CoA production in hypoxic cancer cells reveals substantial contribution from acetate
Source: Cancer Metab. 2014 Dec 11;2:23. doi: 10.1186/2049-3002-2-23 (PMC4322440; doi:10.1186/2049-3002-2-23)
Supplement: Supplementary file 1 — Additional file 1: Figure S1: Percent labeling of acetyl groups from U-13C-glucose (Gluc) and U-13C-glutamine (Gln) in normoxia and hypoxia (1% O2). Acetyl labeling from N-acetyl-glutamate and glutamate at a steady state; analysis of fatty acid labeling gives equivalent results. All data are mean ± SD of N = 3. Figure S2. Calculation of expected palmitate labeling, based on the observation that 20% of palmitate pool is U-13C-labeled, for palmitate oxidation contributing ‘X’ percent to the fraction of AcCoA not derived from glucose or glutamine. Even if X is 13%, significant M + 2 labeling would be expected, which was not observed experimentally (see Figure 3). (PDF 136 KB) [file 40170_2014_140_MOESM1_ESM.pdf]

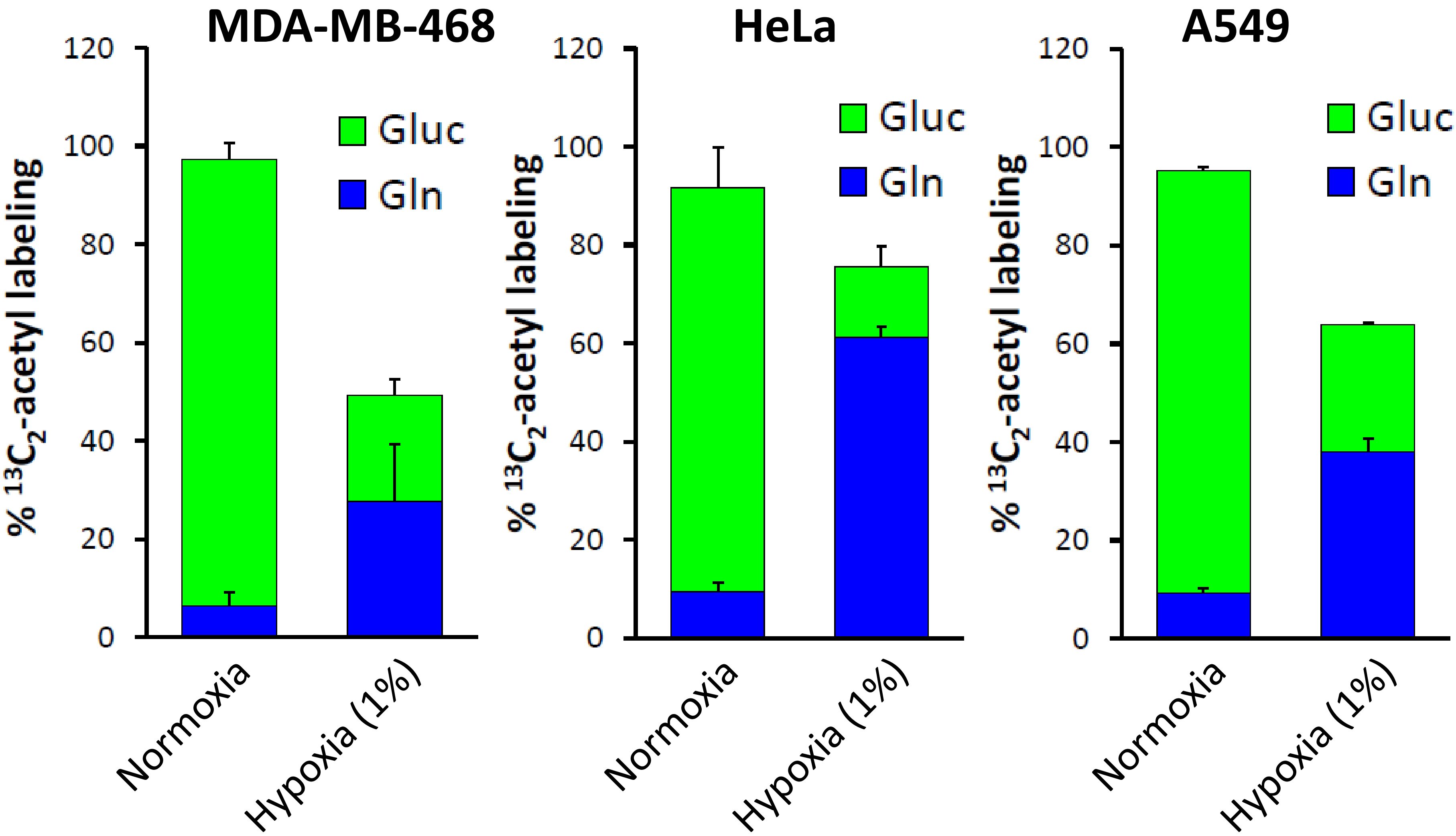

Figure S1. Percent labeling of acetyl groups from U-^{13}C-glucose (Gluc) and U-^{13}C-glutamine (Gln) in normoxia and hypoxia (1% O_2). Acetyl labeling from N-acetyl-glutamate and glutamate at steady-state; analysis of fatty acid labeling gives equivalent results. All data are mean ± SD of *N*=3.

If palmitate pool is 20% labeled, and contributes X % to 'unknown' part of AcCoA pool.

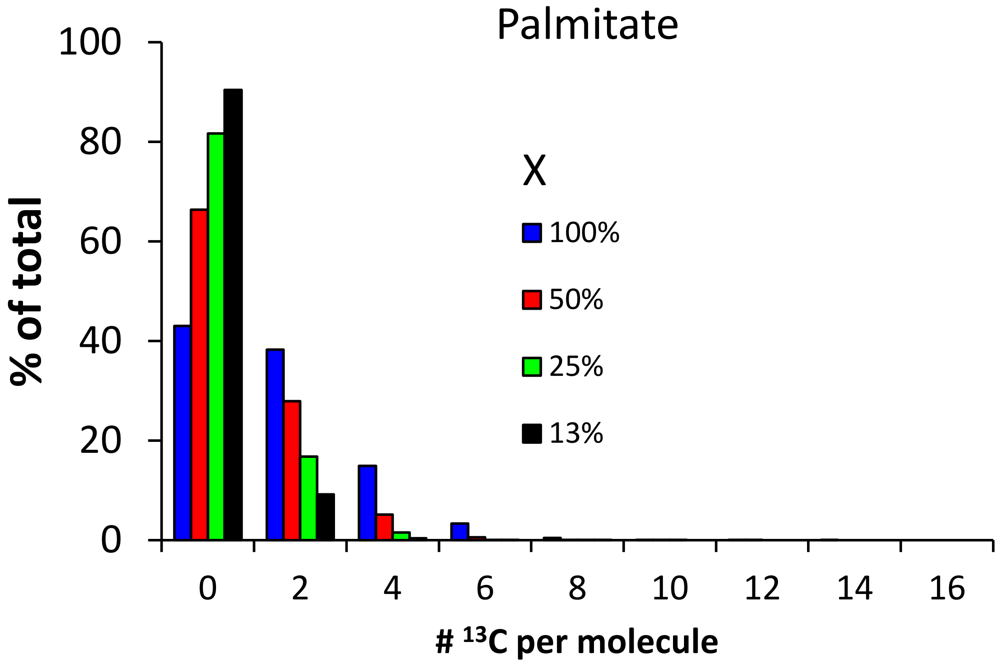

Figure S2. Calculation of expected palmitate labeling, based on the observation that 20% of palmitate pool is $U^{13}C$-labeled, for palmitate oxidation contributing 'X' percent to the fraction of AcCoA not derived from glucose or glutamine. Even if X is13%, significant M^{+2} labeling would be expected, which was not observed experimentally (see Figure 3).
